# Annotations

**Published:** 1910-01-01

**Authors:** 


### Annotations.

### The Lancashire Cotton-worker.

An outspoken criticism of the Lancashire cotton-worker appears in the current issue of the *National Review* from the pen of Dr. Elizabeth Chesser. The article is a remarkably trenchant one, and although it is apparent that the writer lacks sympathy and makes small allowance for human nature, while she bristles with the fervent spirit of a Savonarola, her conclusions are worth serious consideration, and to medical readers at all events highly interesting. Her indictment against the cotton-workers, male and female, is a lengthy one. The infant mortality in factory districts is high, admittedly unnecessarily high, but we question if its excessive ratio is due to the factors Dr. Chesser mentions more than to factors that account for high infantile mortality in other districts. The accusation that the cotton-worker is uneconomical, that his womankind does not know how to keep house, and that he embarks upon matrimony at an early age and without sufficiently providing for his future home and family, is an accusation that can be brought against the majority of our poorer classes. Boy and girl marriages are as common, let us say, in the New Cross or Dalston districts as in Lancashire; the average working-man's wife does not know any better than the average Lancashire lass how to make a palatable, nourishing meal at a minimum cost. In parenthesis we question whether the average English housewife, high or low, knows her duties as she ought, or whether Lancashire deals more with tinned foods than London or Edinburgh. General indictments of this nature are not very telling, especially to those who are acquainted with the conditions under which that large class of folk which is pretty accurately described as "clubbers" live and die. That matters may be a trifle worse in Lancashire is probably correct, and Dr. Chesser's article will do good by drawing attention to the fact, while it is generally interesting as giving the impressions of an expert observer; but that the indictment as a whole against the cotton operative is true remains to be proven.

### The Notification of Births.

The provisions of the Act passed in 1907 for the Notification of Births to medical officers of health were made optional for local authorities, which can adopt the Act or decline to adopt it as they think fit. This has, unfortunately, led to the curious situation, in large towns, of the Act being in force in some localities but not in others; by which anomalous arrangement infants born on one side of a street may be notifiable while those born on the other are not. As a matter of experience it has also been shown that, in districts where the Act has been adopted, as a rule not more than 50 to 70 per cent. of the births are thus notified; and short of prosecutions it is not easy to see how this result is to be improved on. As most practitioners know, the Act imposes the duty of notifying the birth not only upon the father, if residing in the house where the birth occurs, but also upon the attendant at the delivery, whether midwife or doctor. In view of the non-adoption of the Act by many local authorities, prosecutions up to now have been practically unknown. In Kensington, however, it would seem that a more energetic phase is to be entered upon. The medical officer of health has had a circular printed, to be distributed to fathers who fail to notify births, in which he asks for any explanation there may be of the omission. This request is made on the ground that the case is to be laid before the Public Health Committee at its next meeting, and that it will assist the M.O.H. "to know beforehand the reasons for your apparent failure to comply with the . . . Act." Whether this foreshadows legal processes for the recovery of the penalties provided by the Act remains to be seen; but as some of the adjoining boroughs have not adopted the Act it is perhaps doubtful whether courts of summary jurisdiction will prove very sympathetic.

### The Moral of Infantile Mortality.

Unfortunately the questions of motherhood and infant nature are popularly regarded as sentimental rather than scientific, and the consequence of this is that the lives of infants are sacrificed to a sentiment from which statistics show they have urgent need of rescue. An interesting illustration of these facts is to be found in the report of the medical officer of health for Staffordshire. Experience of the homes and mothers of the poorer classes of England has led him to express the view "that it is hopeless to attempt to educate the present race of mothers, and that no real headway will be made until the rising generation of both sexes is systematically taught elementary health principles at school." There is a scientific spirit and a freedom from cant in this which are noteworthy, if only because no other post offers finer opportunities for arriving at the facts than that of medical officer of health. The Board of Education and the London Hospitals are to test by experiment the possibilities of treating school children systematically, and this experiment in London, coupled with the work of medical officers of health in the provinces, of which we have given an example, point to the close alliance of medicine and education in the campaign against disease. That this alliance is a wise one we do not doubt, and the moral which the Staffordshire medical officer has drawn from the figures of infantile mortality in his district is the proof that the sooner that alliance is systematised the better.

